# Effects of divergent selection upon adrenocortical activity on immune traits in pig

**DOI:** 10.1186/s12917-019-1809-9

**Published:** 2019-03-04

**Authors:** Julie Hervé, Elena Terenina, Karine Haurogné, Elodie Bacou, Elizaveta Kulikova, Marie Allard, Yvon Billon, Jean-Marie Bach, Pierre Mormède, Blandine Lieubeau

**Affiliations:** 1 IECM, INRA, Oniris, La Chantrerie, CS 40706, F-44307 Nantes Cedex 3, France; 2 GenPhySE, Université de Toulouse, INRA, INPT, ENVT, F-31326 Castanet-Tolosan, France; 3 Federal Research Center, Institute of Cytology and Genetics, Siberian Branch of Russian Academy of Sciences, Novosibirsk, Russian Federation 630090; 4 GenESI, INRA, Le Magneraud, F-17700 Saint-Pierre-d’Amilly, France

**Keywords:** Stress, Hypothalamic-pituitary-adrenocortical axis, Cortisol, LPS challenge, Genetics, Robustness, Immunity

## Abstract

**Background:**

The sustainability of farming and animal welfare requires the reconsideration of current selection schemes. In particular, implementation of new selection criteria related to animal health and welfare should help to produce more robust animals and to reduce anti-microbial use. The hypothalamo-pituitary-adrenocortical (HPA) axis plays a major role in metabolic regulation and adaptation processes and its activity is strongly influenced by genetic factors. A positive association between HPA axis activity and robustness was recently described. To explore whether selecting pigs upon HPA axis activity could increase their robustness, a divergent selection experiment was carried out in the Large White pig breed. This allowed the generation of low (HPA^lo^) and high (HPA^hi^) responders to adrenocorticotropic hormone administration.

**Results:**

In this study, we compared 23 hematologic and immune parameters of 6-week-old, HPA^lo^ and HPA^hi^ piglets and analysed their response to a low dose of lipopolysaccharide (LPS) two weeks later. At six weeks of age, HPA^hi^ piglets displayed greater red blood cell and leucocyte number including CD8α^+^ γδ cells, cytotoxic T lymphocytes, naive T helper (Th) cells and B lymphocytes as compared to HPA^lo^ individuals. The ability of blood cells to secrete TNFα in response to LPS ex vivo was higher for HPA^hi^ pigs. At week eight, the inflammatory response to the LPS in vivo challenge was poorly affected by the HPA axis activity.

**Conclusions:**

Divergent selection upon HPA axis activity modulated hematologic and immune parameters in 6-week-old pigs, which may confer an advantage to HPA^hi^ pigs at weaning. However, HPA^lo^ and HPA^hi^ piglets did not exhibit major differences in the parameters analysed two weeks later, *i. e.* in 8-week-old pigs. In conclusion, chronic exposure to high cortisol levels in HPA^hi^ pigs does not negatively impact immunity.

**Electronic supplementary material:**

The online version of this article (10.1186/s12917-019-1809-9) contains supplementary material, which is available to authorized users.

## Background

For decades, genetic selection in the pig industry focused on production criteria with deleterious consequences on health and welfare (reviewed in [1]). The sustainability of farming, including animal welfare, requires reconsideration of selection schemes. Implementation of new genetic selection criteria in relation to pig health could help reducing antibiotic use, which is urgently needed to address public health concerns [2, 3] and is strongly encouraged by the European Commission (for details, see [4]). In particular, selecting pigs for robustness could increase animal resilience to pathogens.

A positive association between HPA axis activity and robustness was previously described [5]. The HPA axis plays a major role in metabolic regulation and adaptation processes and its activity is strongly influenced by genetic factors [6, 7]. Together with the autonomous nervous system, it helps the organism to cope with threats and infections. Cortisol is the main terminal effector of the HPA axis in humans and pigs. Its secretion by adrenal glands is controlled by the release of the corticotropin-releasing factor by the hypothalamus, which induces subsequent adrenocorticotropic hormone (ACTH) secretion by the anterior pituitary gland. Adrenal gland sensitivity to ACTH was demonstrated to differ largely among individuals while being a highly heritable trait [8]. We hypothesized that selecting pigs upon the adrenal response to ACTH could increase their robustness. To explore this strategy, a divergent selection experiment was carried out in the Large White pig breed based on plasma cortisol levels measured one hour after ACTH administration [8, 9]. In second generation offsprings, we showed that high- (HPA^hi^) and low- (HPA^lo^) responders to ACTH stimulation differ in their ability to cope with an acute social stress, with HPA^hi^ piglets appearing more resilient [10]. We also highlighted some differences in immune traits between the two populations of pigs. Indeed, HPA^hi^ pigs displayed greater CD4^+^ T cell counts and their blood cells secreted higher amounts of TNFα after LPS stimulation.

In third generation offsprings, the post-ACTH plasma cortisol level was found to be 2.16 times higher in the HPA^hi^ line than in the HPA^lo^ line [9]. In the current study, we aimed to compare hematologic and immune parameters of 16 HPA^hi^ and 16 HPA^lo^ piglets belonging to this third generation of divergent selection at six (experiment 1) and eight (experiment 2) weeks of age.

## Results

### Effects of HPA axis activity on immune traits in pigs

**Fig. 1:**
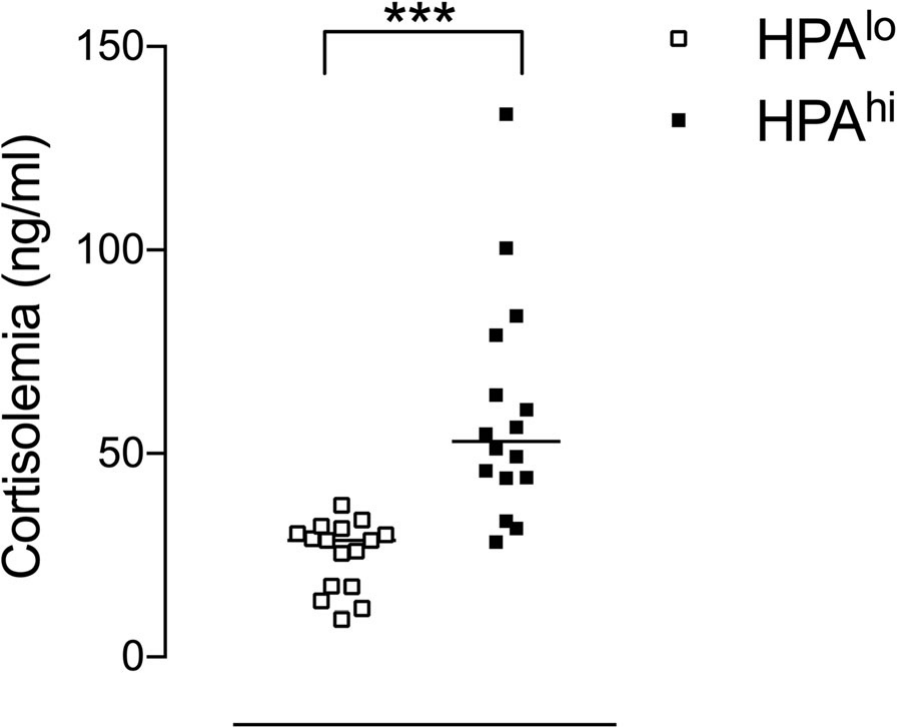
Steady-state cortisolemia in HPA^lo^ and HPA^hi^ piglets. Plasma cortisol levels were measured in 6-week-old HPA^lo^ (empty squares) and HPA^hi^ (black squares) piglets (*n* = 16 per group). Each point represents an individual and medians are shown. Stars denote statistical significance (*p* < 0.001)

In experiment 1, hematologic and immune parameters of 6-week-old HPA^lo^ (*n* = 16) and HPA^hi^ (*n* = 16) pigs were assessed. As expected, pigs exhibited significantly different steady-state cortisolemia with HPA^hi^ piglets secreting twice as much cortisol than HPA^lo^ pigs (Fig. 1).

**Fig. 2:**
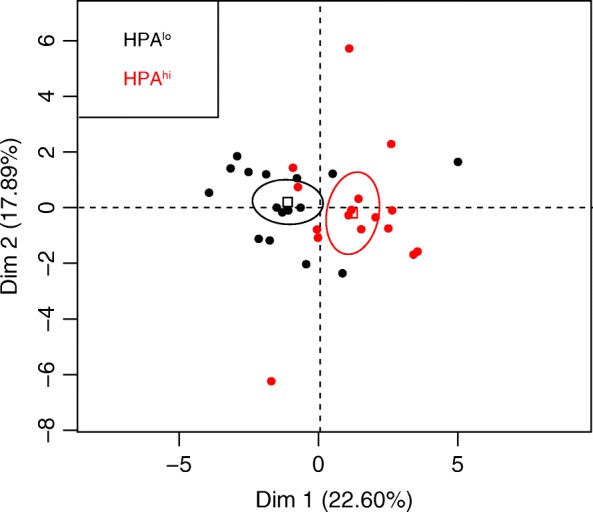
Principal component analysis: individuals factor map. PCA was performed using values obtained for 20 variables measured in 6-week-old HPA^lo^ (black dots) and HPA^hi^ (red dots) pigs (*n* = 16 per group). Each point represents a single individual in the first two dimensions of the PCA

To get an overview of our data, we first performed a Principal Component Analysis (PCA) on raw data. Individuals are mapped along the first two dimensions of the PCA (Fig. 2), while contributions of individual variables to the first two dimensions are presented in Additional file 1: Figure S1 and Additional file 2: Table S1. The first axis was mainly determined by white blood cell parameters (CD4^+^ and CD8α^hi^ T cells), while the second axis mainly resulted from red blood cell parameters (RBC, HGB and HCT). HPA^lo^ and HPA^hi^ piglet populations appeared clearly separated, as shown by their confidence ellipses, especially along the “white blood cell” axis.

**Fig. 3:**
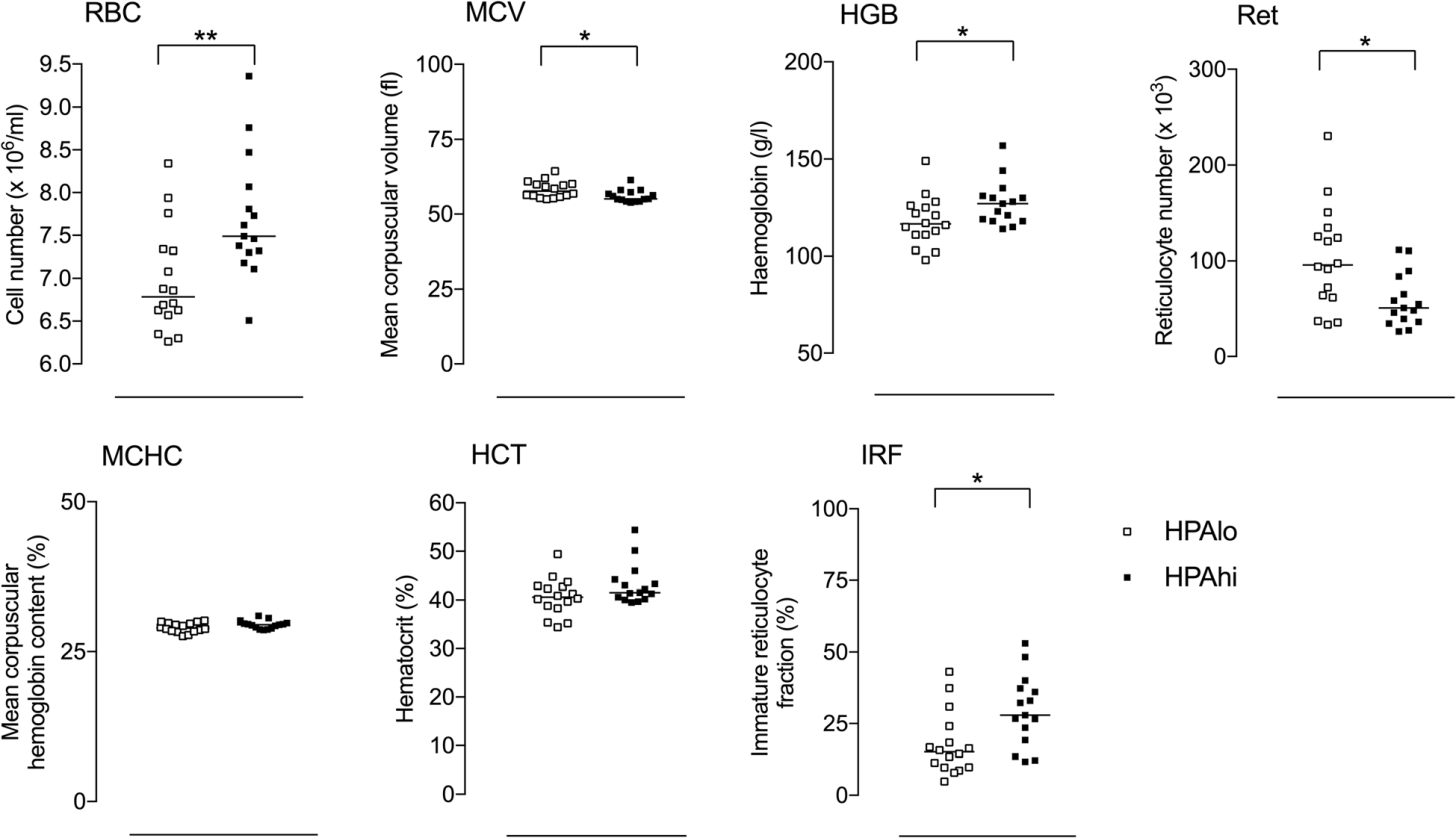
Red blood cell parameters in HPA^lo^ and HPA^hi^ pigs. Red blood cell parameters (RBC, HCT, MCV, HGB, MCHC, Ret and IRF) were measured in EDTA-treated blood of HPA^lo^ (empty squares) and HPA^hi^ (black squares) piglets (*n* = 16 per group) using the Procyte™ hematimeter. Each point represents a single piglet. Stars denote statistical significance (*p < 0.05; ** *p* < 0.01)

While divergent selection did not modify platelet counts (*data not shown*), some red and white blood cell parameters appeared to be affected by selection of pigs upon HPA axis activity. As shown in Fig. 3, among seven red blood cell parameters measured, five significantly differed between HPA^lo^ and HPA^hi^ pigs, namely red blood cell count (RBC), mean corpuscular volume (MCV), haemoglobin (HGB), reticulocyte count (Ret) and immature reticulocyte fraction (IRF). While RBC, HGB and IRF were higher in HPA^hi^ pigs, MCV and Ret were lower as compared to HPA^lo^ individuals.

**Fig. 4:**
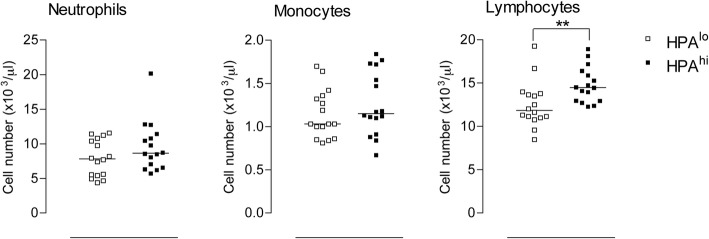
White blood cell counts of both lines of pigs. Neutrophil, monocyte and lymphocyte cell counts were measured in EDTA-treated blood of HPA^lo^ (empty squares) and HPA^hi^ (black squares) piglets (*n* = 16 per group) using the Procyte™ hematimeter. Each point represents a single piglet. Stars denote statistical significance (** *p* < 0.01)

**Fig. 5:**
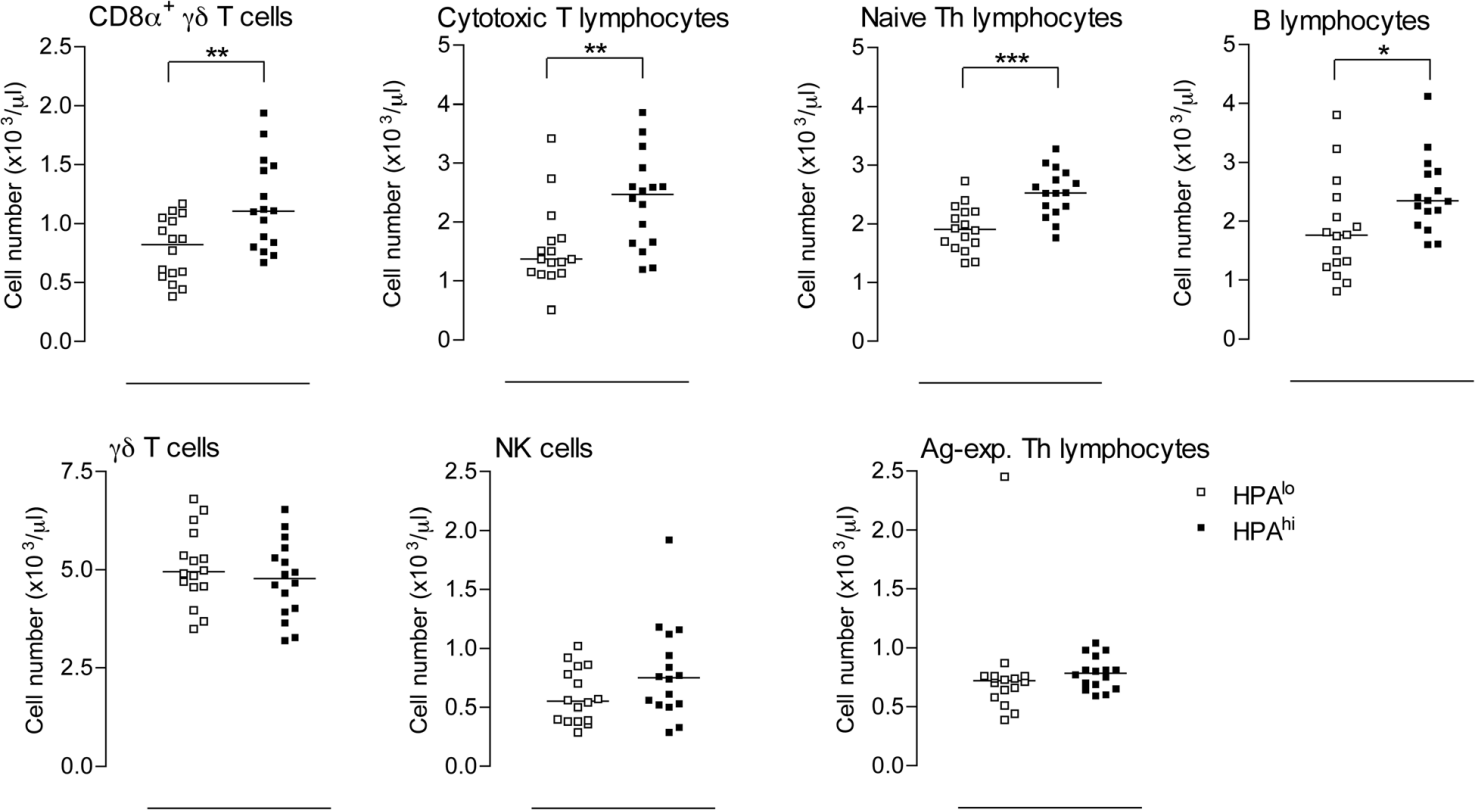
Specific variations in lymphocyte subpopulations of HPA^lo^ and HPA^hi^ piglets. Lymphocyte subpopulations were characterized in EDTA-treated blood of 6-week old HPA^lo^ (empty squares) and HPA^hi^ (black squares) piglets (*n* = 16 per group) by multicolor staining using flow cytometry. Seven populations were identified: B lymphocytes (CD3^−^ CD21^+^), naive CD4^+^ Th cells (CD3^+^ CD4^+^), antigen-experienced Th cells (CD3^+^ CD4^+^ CD8α^+^), CD8α^−^ γδ T cells (CD3^+^ CD4^−^ CD8α^−^), CD8α^+^ γδ T cells (CD3^+^ CD4^−^ CD8α^med^), CD8α^hi^ cytotoxic T lymphocytes (CD3^+^ CD4^−^ CD8α^hi^) and NK cells (CD3^−^ CD4^−^ CD8α^med^); gating strategy is shown in Additional file 3: Figure S2. Each point represents a single piglet. Stars denote statistical significance (*p < 0.05; ** *p* < 0.01; ***p < 0.001)

Concerning white blood cell parameters, leucocyte count was significantly higher in HPA^hi^ as compared to HPA^lo^ pigs at six weeks of age (*p* < 0.01, data not shown). This difference in leucocyte counts essentially relied on lymphocyte counts since neutrophil and monocyte counts did not vary significantly between the two groups (Fig. 4). To better characterise the difference in lymphocyte numbers, we used flow cytometry, which allowed the discrimination of seven lymphocyte subpopulations (Additional file 3: Figure S2 and not shown for B cells). Four lymphocyte subpopulations appeared higher in HPA^hi^ as compared to HPA^lo^ individuals: CD8 α^+^ γδ T cells, CD8α^hi^ cytotoxic T lymphocytes, naive CD4^+^ Th cells and CD21^+^ B lymphocytes (Fig. 5). Of note, CD8α^−^ γδ cell, NK cell and antigen-experienced Th lymphocyte numbers were similar between the two groups.

**Fig. 6:**
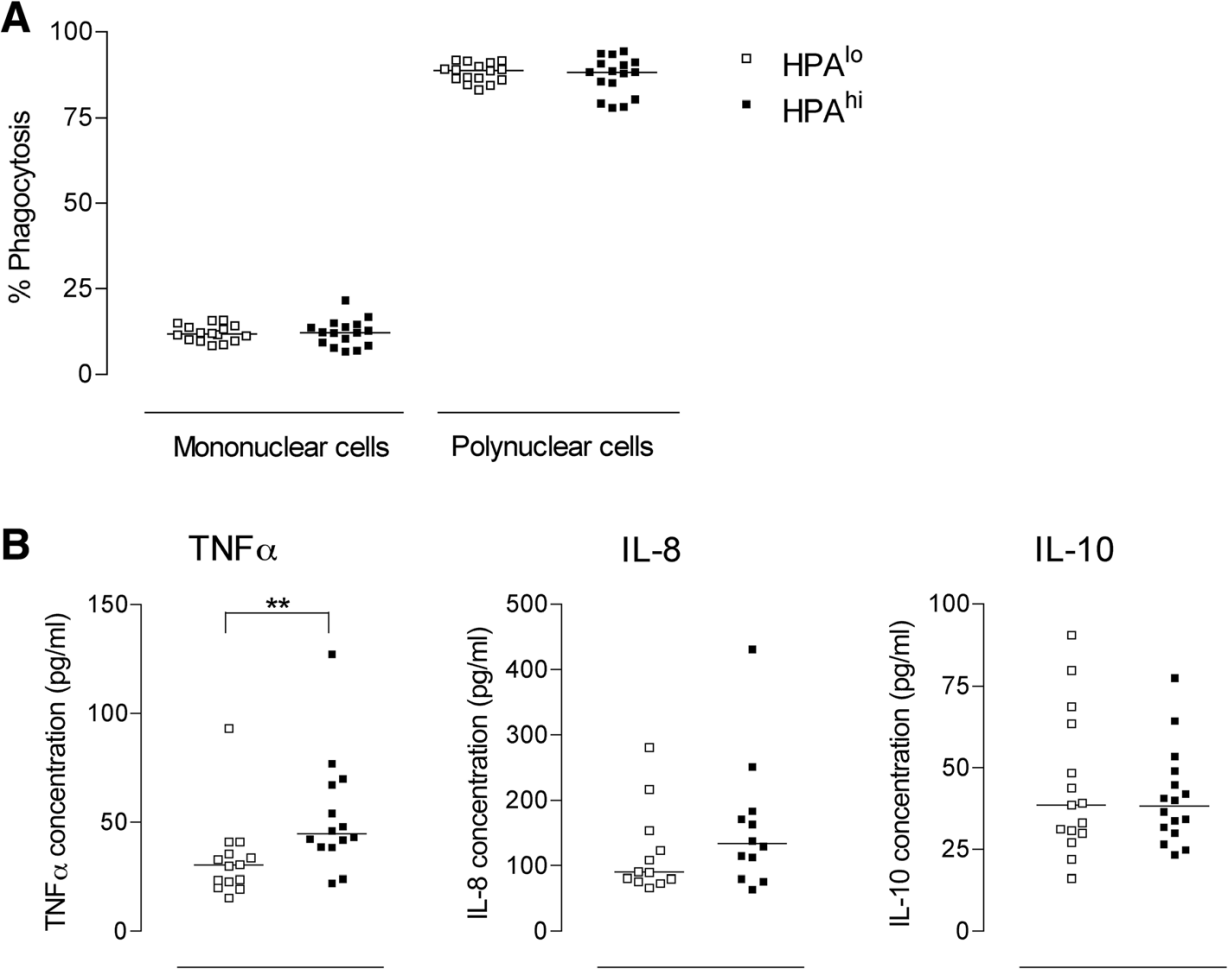
Functional ability of circulating immune cells in both lines of pigs. Phagocytosis (**a**) and cytokine secretion (**b**) were evaluated on heparin-treated blood of 6-week old HPA^lo^ (empty squares) and HPA^hi^ (black squares) pigs (*n* = 16 per group). Each point represents a single piglet. **a**, Phagocytosis of fluorescent *E. coli*. Bacteria by mononuclear and polynuclear cells was analysed by flow cytometry. **b**, Cytokine secretion by whole blood cells was evaluated after an ex vivo stimulation for 18-h with 10 ng/ml LPS. The amount of TNFα, IL-8 and IL-10 in the supernatants was measured by ELISA. Stars denote statistical significance (** *p* < 0.01)

To investigate whether selection upon HPA axis strength also modulated leucocyte functions in 6-week-old pigs, we performed ex-vivo phagocytosis and whole blood assays (Fig. 6). Mononuclear and polynuclear cell phagocytosis were similar for HPA^lo^ and HPA^hi^ pigs (Fig. 6a). Concerning LPS-induced cytokine secretion by blood cells, while IL-8 and IL-10 were not different between the two groups, TNFα secretion from cells derived from pigs with strong HPA axis activity was significantly higher (Fig. 6b).

### Effects of HPA axis activity on acute response to LPS in 8-week-old pigs

**Fig. 7:**
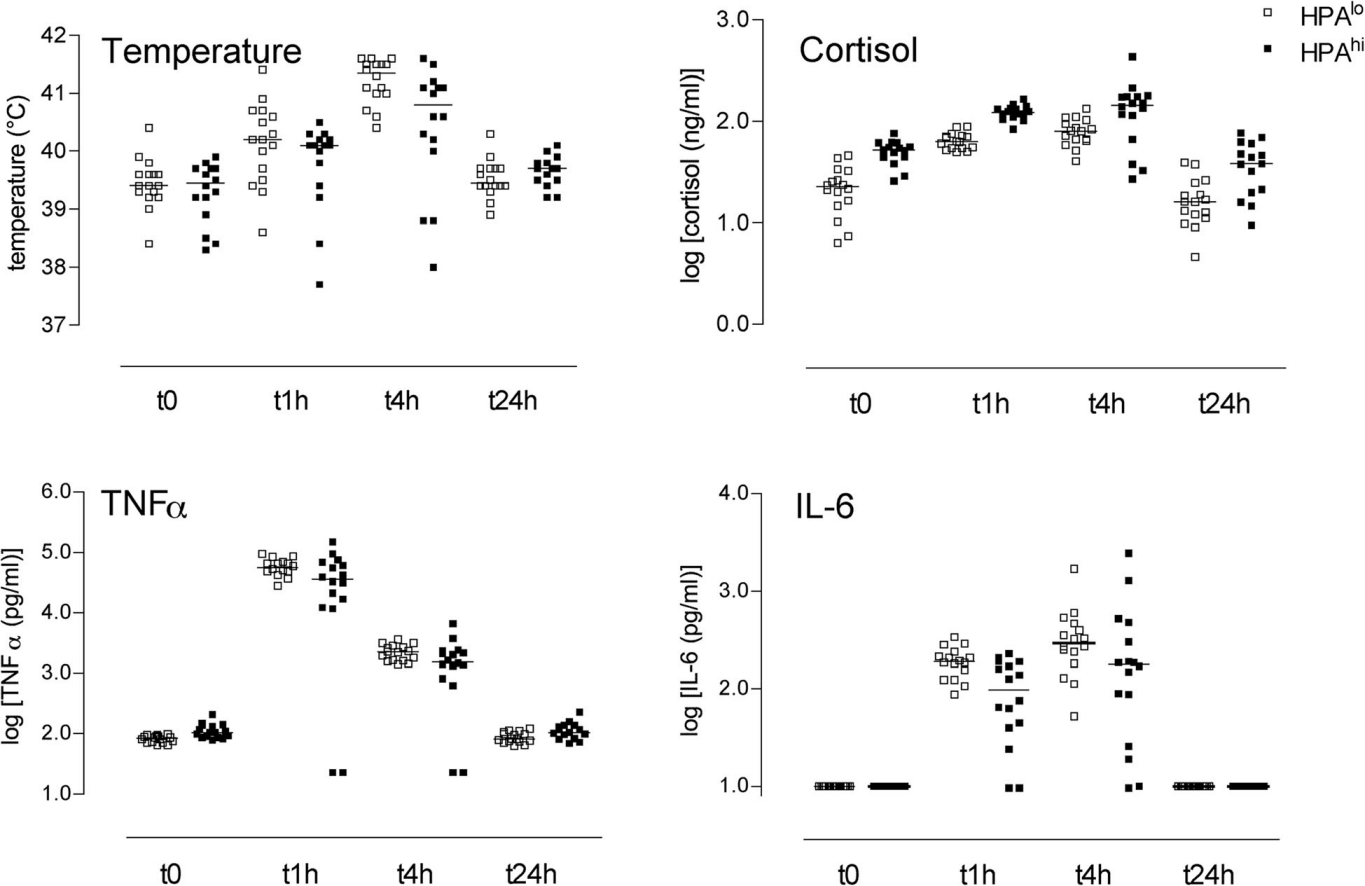
Effect of LPS injection on body temperature, cortisol, TNFα and IL-6 plasma levels in 8-week-old pigs. Blood was collected in heparin-coated tubes from 8-week old HPA^lo^ (empty squares, *n* = 16) and HPA^hi^ (black squares, *n* = 15) pigs just before (t0) and 1, 4 and 24 h following the *i.m.* injection of LPS. At each time point, temperature was measured into the ear. Cortisol, TNFα and IL-6 levels were measured in plasma samples. Each point represents a single piglet. See Table 1 for statistical analysis

Experiment 2 was performed on the same animals two weeks later. We administered a low dose of LPS (15 μg/kg) and investigated its consequences on several parameters focusing on short-term effects, i.e. within 24 h after intramuscular LPS injection. While this protocol is not known to induce sickness behaviour (vomiting, diarrhoea and/or somnolence), one pig from the HPA^hi^ line died between 4 and 24 h after LPS injection and was hence excluded from the analysis. In pigs from both populations, LPS induced a rise in tympanic temperature, although less intense in HPA^hi^ pigs (Fig. 7, Table 1).

**Table 1:** Results of the statistical analysis of the in vivo experiment (*p*-values)

Parameters	HPA activity	Time	Interaction
Tympanic temperature	0.077	<2e-16	0.026
White blood cell counts (log)	0.052	<2e-16	0.015
Lymphocyte to granulocyte ratio (log)	0.92	<2e-16	0.64
Red cells	0.069	1.11e-12	0.503
Cortisol (log)	4.64e-07	<2e-16	0.052
Blood glucose	0.329	<2e-16	0.080
Free fatty acids (square root)	0.064	2.11e-12	0.236
Bilirubin	0.31	<2e-16	0.167
TNFα (log)	0.13	<2e-16	2.46e-4
IL-6 (log)	0.013	<2e-16	2.38e-4

For each parameter, the effects of HPA activity, time and their interaction were tested in a two-way analysis of variance with repeated measures for the time factor

Also, cortisolemia rapidly increased after LPS injection with a peak at 4 h (Fig. 7). As expected, HPA^hi^ pigs released significantly greater amounts of cortisol at every time point after LPS challenge than HPA^lo^ pigs did (Table 1). In nearly all HPA^lo^ and HPA^hi^ individuals, stress response to LPS was accompanied by a decrease in glycaemia and an increase in free-fatty acid release and bilirubinaemia. The profiles of white blood cell count and lymphocyte-to-granulocyte ratio in response to LPS were similar between pigs from both populations (Table 1, *p* = 0.052 and 0.92 respectively).

A transcriptomic analysis was also conducted to monitor divergent selection effects on the expression of 34 genes modulated by LPS. Among them, 23 genes were up-regulated at t1 h, 13 genes were down-regulated at t1 h, 1 gene was up-regulated at t4 h, 21 genes were down-regulated at t4 h and 4 genes were down-regulated at t24 h (Additional file 4: Table S2). No differences in LPS-modulated transcript levels were observed between the two populations of pigs at any time point (*data not shown*). Finally, plasma TNFα and IL-6 levels after LPS administration were measured. Typical secretion curves were obtained, peaking at t1 h and t4 h, respectively (Fig. 7). While TNFα levels did not differ between individuals from the two populations, plasma IL-6 levels were higher in HPA^lo^ as compared to HPA^hi^ pigs (Table 1, *p* = 0.013). Strikingly, two full-sib individuals of the HPA^hi^ group behaved differently and responded poorly to the LPS challenge.

## Discussion

In this study, we aimed to document on the effects of divergent genetic selection upon HPA axis activity on blood cell counts and functions by comparing data obtained from 16 HPA^lo^ and 16 HPA^hi^ pigs in basal conditions (experiment 1) and after LPS challenge (experiment 2), respectively at 6 and 8 weeks of age.

At 6 weeks of age, HPA^hi^ pigs displayed a higher red blood cell count and haemoglobin content, and a lower mean corpuscular volume. HPA^hi^ individuals also exhibited a lower reticulocyte number associated with a higher immature reticulocyte fraction, which reflects the proportion of younger reticulocytes [11]. The higher red blood cell count in association with a greater immature reticulocyte fraction suggests a stronger erythropoietin activity in HPA^hi^ pigs, which could increase the oxygen carrying capacity of blood (reviewed in [12]). Interestingly, the plasma cortisol level was recently shown to be positively correlated to the erythropoietin (EPO) level in healthy human volunteers [13]. EPO is a glycoprotein controlling early events of erythropoiesis in the bone marrow thereby increasing the number of red blood cells [14]. In this context, it could be interesting to measure serum EPO levels in piglets from both populations. Whether the higher oxygen carrying capacity of blood could boost HPA^hi^ piglet growth and performance is currently under investigation.

While moderate and short-term elevations of circulating glucocorticoids (GCs) may promote immunity, high and long-term elevations of circulating GCs were rather shown to be immunosuppressive (reviewed in [15]). In particular, GCs induce lymphocyte sequestration into tissues thereby reducing their number in the circulation [16, 17]. While studying diurnal rhythm effects on blood cell numbers in pigs, Engert et al. recently underscored negative associations between plasma cortisol levels and most lymphocyte subtypes [18]. However, in our study, despite a higher cortisolemia, 6-week-old HPA^hi^ piglets displayed a higher leucocyte count, which mainly relied on B lymphocytes, naive Th lymphocytes, cytotoxic T lymphocytes and CD8α^+^ γδ T cells. Whether this increase in circulating lymphocytes may foster the immune capacity of HPA pigs is still unknown. We next evaluated the “innate immune response signature” of HPA^lo^ and HPA^hi^ piglets by assessing LPS-induced TNFα, IL-8 and IL-10 secretion using whole blood assays (WBA) and phagocytosis of opsonized *E. coli* bacteria. While most functions were preserved in HPA^hi^ piglets, TNFα secretion was significantly higher in 6-week-old HPA^hi^ pigs as compared to HPA^lo^ individuals, confirming our previous results obtained in animals from the second generation of selection [10]. In this previous experiment, HPA^hi^ piglets exhibited a higher naive Th lymphocyte number and greater LPS-induced TNFα secretion than did HPA^lo^ pigs did. Interestingly, after another round of divergent genetic selection, we also evidenced an increase in three other lymphocyte subpopulations, namely cytotoxic T lymphocytes, CD8α^+^ γδ T cells and B lymphocytes, in 6-week-old, HPA^hi^ pigs. Given their immaturity, young piglets are more fragile, weaning being the most critical period of their lives. From the first experiment, we presume that HPA^hi^ piglets might be better prepared to cope with stress including exposure to pathogens in the first months of their life.

We next evaluated metabolic and immune responses to in vivo LPS challenge at week 8. In both lines, LPS induced activation of the HPA axis, as shown by the elevation of plasma cortisol levels following injection, confirming previous observations in unselected pigs [19]. The LPS-induced metabolic changes also consisted in an increase in free fatty acid release and bilirubinaemia associated to a decrease in glycaemia, probably due to increased glycolysis and decreased neoglucogenesis. No difference between HPA^lo^ and HPA^hi^ pigs was evidenced for most immune parameters observed, except for plasma IL-6 levels, which were higher in the HPA^lo^ line. Thus, the divergent genetic selection program poorly affected the LPS response of 8-week-old pigs, which is in agreement with our previous work. Indeed, in unselected Large White pigs, responses to LPS were not influenced by adrenal gland reactivity as assessed by the cortisol response to ACTH.

Interestingly, in 8-week-old pigs, the difference in baseline blood cell counts previously observed between the two lines of pigs had disappeared (data not shown). Chronic exposure to high cortisol levels is known to induce GC resistance as an adaptive process, notably through down-regulation of glucocorticoid receptors (GR) expression. Age-related effects on lymphocyte cortisol sensitivity were also documented in pigs [20].

In addition, in the case of this divergent selection experiment, exposure to differential GC levels might have led to select individuals with different intrinsic sensitivity to GC. A Genome Wide Association Study conducted on siblings actually revealed that the selection process segregated two alleles of the *NR3C1* gene, which encodes GR [9]. HPA^lo^ pigs, in contrast to HPA^hi^ pigs, carry the previously described mutated form of swine *NR3C1* gene [21]. This allele encodes an A610V substitution in the ligand-binding domain of GR, which induces a gain of function of the receptor leading to GC hypersensitivity. Pigs carrying this mutation did not exhibit major phenotypic differences in growth and body composition, but they displayed a transient lower leucocyte number compared to the carriers of the wild type gene [22]. In this context, we can wonder whether the overall exposure of HPA^lo^ and HPA^hi^ pigs to GC is different.

## Conclusion

In this study, we confirmed that young HPA^hi^ piglets exhibit greater leucocyte counts and TNFα secretion in whole-blood assay than HPA^lo^ pigs, but these differences decreased with age. We also demonstrated that chronic exposure to high cortisol levels in HPA^hi^ pigs does not negatively impact immunity.

## Methods

### Animals and experimental design

All experimental procedures were carried out in accordance with the EU directive 2010/63 for animal experiments and submitted to and approved by the C2EA 84 Poitou-Charentes Region ethics committee (decision #CE2013–1, 2013.01.21).

A divergent genetic selection experiment was conducted in purebred Large White pigs by selecting, at each generation, founders with extreme cortisolemia following ACTH stimulation as an indicator of HPA axis activity. This allowed the generation of pigs low (HPA^lo^) and high (HPA^hi^) responders to ACTH injection [9]], which were all born and raised at the INRA experimental unit of Le Magneraud (UE1372 GenESI) until euthanasia by lethal electronarcosis.

This study involved blood samples collected from 32 healthy female pigs belonging to the third generation of divergent selection (16 HPA^lo^ and 16 HPA^hi^ pigs). In each population, the 16 pigs were offspring of 8 sows mated with 8 different barrows, constituting 8 pairs of full-sibs.

After weaning at 4 weeks, pigs were housed by groups of 20 to 22 individuals per pen in controlled ambient conditions (27 °C, 12 h/12 h light/dark cycle) with unrestricted access to food and water. Two experiments were conducted. At week 6, hematologic and immune parameters were analysed on blood samples collected from 9 to 10 *a.m* (Experiment 1)*.* Two weeks later, a kinetic response to intra-muscular LPS injection (15 μg/kg in the neck muscles) was performed on the same animals with blood samples collected just before (t0, between 9 and 10 *a.m.*) and 1, 4 and 24 h after LPS administration as previously described [19] (Experiment 2).

In both experiments, blood was collected from the jugular vein of pigs slightly maintained in a supine position by experienced staff, in less than 30 s per pig as previously described, with a total volume collected never exceeding 25 ml per pig [23]. All blood samples were processed by blinded investigators.

### Complete blood cell counts

Complete blood cell counts (CBC) were determined from EDTA-treated blood samples using the clinical-grade Procyte Dx haematology analyser (IDEXX, Saint-Denis, France). Red blood cell parameters included red blood cell count (RBC), haematocrit (HCT), haemoglobin (HGB), mean corpuscular volume (MCV), mean corpuscular haemoglobin concentration (MCHC), reticulocyte count (Ret) and immature reticulocyte fraction (IRF). CBC also included platelet count as well as white blood cell parameters: white blood cell count (WBC), neutrophil, lymphocyte and monocyte counts.

### Analysis of lymphocyte subpopulations

Lymphocyte subpopulations were identified from EDTA-treated blood samples by two types of staining using monoclonal antibodies: (1) FITC-conjugated anti-pig CD4 (Acrys Antibodies, Herford, Germany), PE-conjugated anti-pig CD8α (BD Biosciences) and PerCP-conjugated anti-pig CD3 (Bio-Techne, Lille, France) antibodies, or (2) PE-conjugated anti-pig CD21 (Acrys Antibodies) and PerCP-conjugated anti-pig CD3 in the dark for 15 min at room temperature. Erythrocytes were removed using a lysis solution (BD Biosciences, Le Pont de Claix, France). The DNA marker DRAQ5™ (1 μM final; Biostatus, Leicestershire, UK) was added to identify nucleated cells, then samples were analysed with a FACS Aria flow cytometer (BD Biosciences) and data computed using the FlowJo software (FlowJo, Ashland, Oregon). Staining allowed the identification of seven different lymphocyte subpopulations as previously described [18]: B lymphocytes (CD3^−^ CD21^+^), naive CD4^+^ Th cells (CD3^+^ CD4^+^), antigen-experienced Th cells (CD3^+^ CD4^+^ CD8α^+^), CD8α^−^ γδ T cells (CD3^+^ CD4^−^ CD8α^−^), CD8α^+^ γδ T cells (CD3^+^ CD4^−^ CD8α^med^), CD8α^hi^ cytotoxic T lymphocytes (CD3^+^ CD4^−^ CD8α^hi^) and NK cells (CD3^−^ CD4^−^ CD8α^med^). The cell count for each specific subpopulation was then determined as the product of the percentage of this specific subpopulation in the DRAQ5^+^ FSC/SSC white blood cell gate by the absolute number of white blood cells obtained from the Procyte analyser.

### Phagocytosis

Ex vivo phagocytosis was assessed using the Phagotest™ Kit (BD Biosciences) according to manufacturer’s instructions. Briefly, heparinized blood samples were incubated for 10 min at 37 °C with opsonized FITC-labelled *E. coli* bacteria. For each analysis, ice-incubated negative controls were included. Samples were immediately analysed by flow cytometry using FSC/SSC dot plot to discriminate granulocytes from mononuclear cells.

### Whole blood assay (WBA)

Heparinized blood samples were five-fold diluted in RPMI 1640 medium supplemented with 2 mM L-glutamine, 100 IU/ml penicillin and 100 mg/ml streptomycin and stimulated in duplicate using 10 ng/ml *Escherichia coli* O111:B4 LPS (Sigma-Aldrich, St-Quentin-Fallavier, France). After an 18-h incubation at 37 °C, in 5% CO2, 95% relative humidity, supernatants were collected, centrifuged at 500 g for 5 min at 4 °C and stored at − 80 °C before cytokine concentration measurements. Porcine TNFα, IL-8 and IL-10 were quantified by ELISA with respective detection limits of 15, 62 and 12 pg/ml (Bio-Techne). For statistical analyses, we attributed to undetected samples half of the corresponding threshold value.

### Kinetic response to LPS

Cortisol, glucose and free fatty acid plasma levels were assessed after LPS administration as previously described [23].

Plasma cytokine levels in response to LPS injection were quantified in heparin-treated samples by ELISA (porcine IL-6 and TNFα, Bio-Techne).

In order to investigate divergent selection effects on gene expression response to LPS, we tested expression of 34 genes modulated by LPS. These genes were provided by the literature and from our own data ([23] and unpublished data). Gene expression analysis was performed as previously described [23]. Total RNA extraction was done using the Nucleospin RNA Blood kit (Macherey-Nagel, Hoerdt, France) followed by DNase treatment. The quality of each RNA preparation was verified through the Bioanalyser Agilent 2100 (Agilent Technologies, Massy, France) and low-quality RNA samples were discarded (RIN < 8). One μg of total RNA was reverse-transcribed. The transferrin receptor and glutamyl-prolyl-tRNA synthetase genes were used as housekeeping genes, according to previous data. Pre-amplified samples were analysed with a 96 × 96 Dynamic Array™ IFC (Fluidigm) following the protocol defined by Spurgeon et al. [24]. Each gene was tested twice for each sample. Four dilution points containing a pool of all samples were used to determine PCR efficiency. Data were analysed using BioMark Gene Expression Data Analysis software (Fluidigm) to obtain Ct values. The Pfaffl method was applied to compute the relative expression of each gene [25]. A linear model was performed to reveal the genes differentially expressed according to the population (high, low) and the time point (0, 1, 4, 24 h):

$$ {expr}_i={\beta}_0+{\beta}_1t+{\beta}_2{population}_i+{\beta}_3{t}^{\ast }{population}_i+{\varepsilon}_i $$

### Statistical analysis

A PCA was performed on raw data obtained on 6-week-old piglets using the FactoMineR package in the R software (version 3.2.1, R Core Team, 2016) [26]. Redundant parameters were excluded from the analysis, i.e. MCV, MCHC, % Ret, WBC and lymphocyte counts. The first two components explained more than 40% of the total inertia and each contributed to more than 15% of the total variance.

Univariate statistical analyses for experiment 1 were made using Graph Pad Prism (version 6.0). For each parameter measured, 16 HPA^lo^ and 16 HPA^hi^ pigs were compared using non-parametric Mann-Whitney tests; statistical significance appears as stars on charts (*: *p* < 0.05; **: *p* < 0.01; ***: *p* < 0.001); each point represents a single animal and lines denote medians.

For experiment 2, cortisol, TNFα, IL-6, white blood cells and platelets data were transformed into their logarithmic scores for normalisation. The square root transformation was used for NEFA (non-esterified fatty acids) levels [19]. For each parameter, the effect of HPA activity, time, and their interaction was tested in a two-way analysis of variance with repeated measures for the time factor. The effects of LPS on tympanic temperature, cortisolemia, plasma TNFα and IL-6 levels were graphed, each point representing a single pig.

## Additional files

Additional file 1: **Figure S1.** PCA variables’ correlogram. Correlations of each variable to the first two dimensions of the PCA are shown. CTL, CD8α^hi^ cytotoxic T lymphocytes. (PDF 129 kb)

Additional file 2: **Table S1.** Variable’s contributions to the first two dimensions of the PCA. Variables that contributed to more than 10% to one axis are underlined. (PDF 35 kb)

Additional file 3: **Figure S2.** Gating strategy for non B-lymphocyte subsets analysis. Representative flow cytometry profile is shown. Nucleated single cells were identified as DRAQ5-positive cells. Among SSC-A^lo^ cells, CD4/CD8α/CD3 co-staining allowed the determination of 6 lymphocyte subsets as mentioned on the plots. (PDF 935 kb)

Additional file 4: **Table S2.** List of genes whose expression was significantly modulated after LPS injection compared to their t0 level. (PDF 43 kb)

## Abbreviations

ACTH: Adrenocorticotropic hormone
CBC: Complete blood cell counts
EPO: Erythropoietin
GC: Glucocorticoids
GR: Glucocorticoid receptors
HCT: Hematocrit
HGB: Haemoglobin
HPA: Hypothalamo-pituitary-adrenocortical
IRF: Immature reticulocyte fraction
LPS: Lipopolysaccharide
MCHC: Mean corpuscular hemoglobin concentration
MCV: Mean corpuscular volume
NEFA: Non-esterified fatty acids
PCA: Principal component analysis
RBC: Red blood cell count
Ret: Reticulocyte count
Th: Helper T cell
WBA: Whole-blood assay
WBC: White blood cell count
