# Supplementary figures and images for: Effects of divergent selection upon adrenocortical activity on immune traits in pig
Source: BMC Vet Res. 2019 Mar 4;15:71. doi: 10.1186/s12917-019-1809-9 (PMC6398250; doi:10.1186/s12917-019-1809-9)

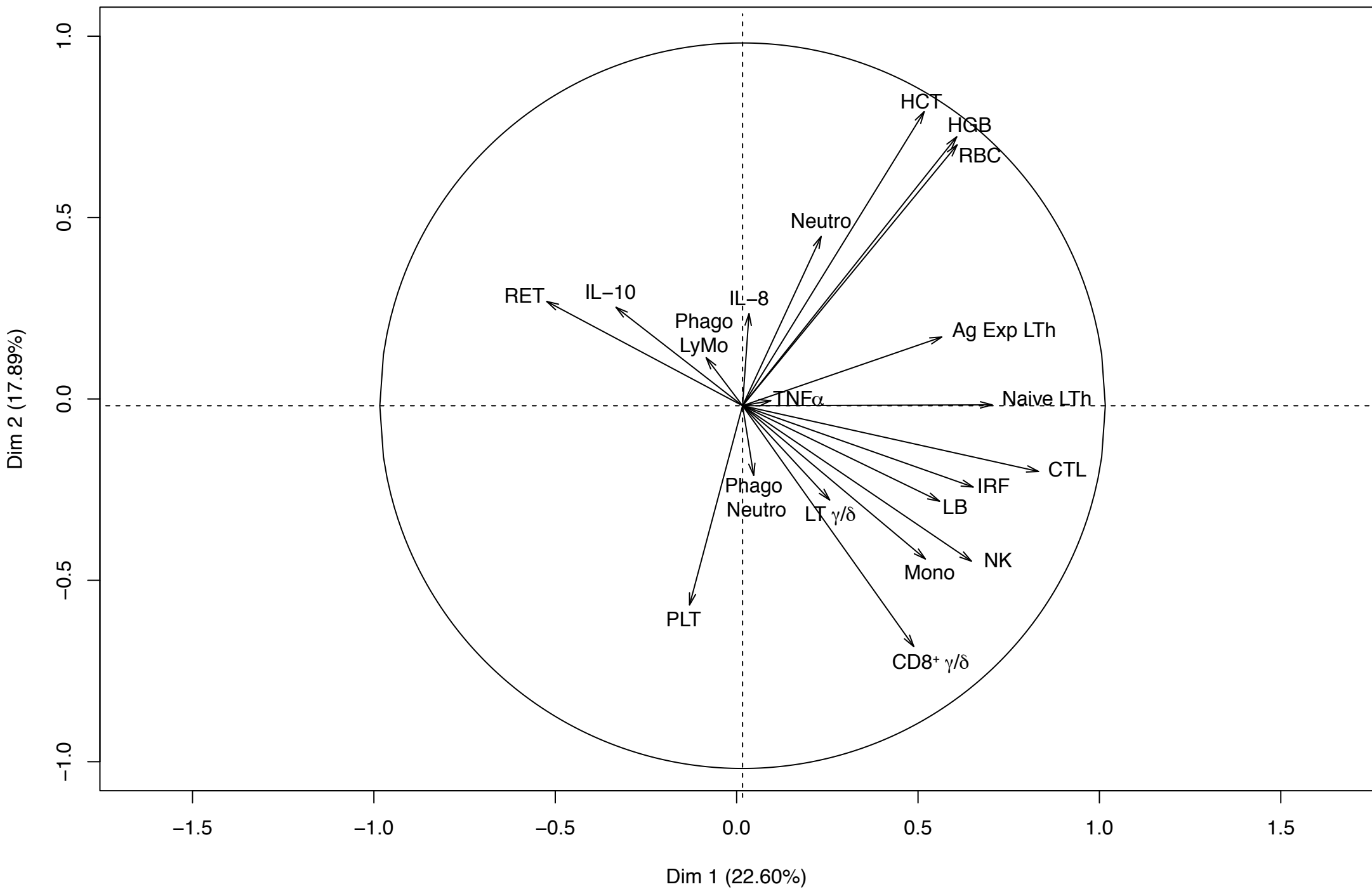

Supplement: Supplementary file 1 — Figure S1. PCA variables’ correlogram. Correlations of each variable to the first two dimensions of the PCA are shown. CTL, CD8αhi cytotoxic T lymphocytes. (PDF 129 kb) [file 12917_2019_1809_MOESM1_ESM.pdf]

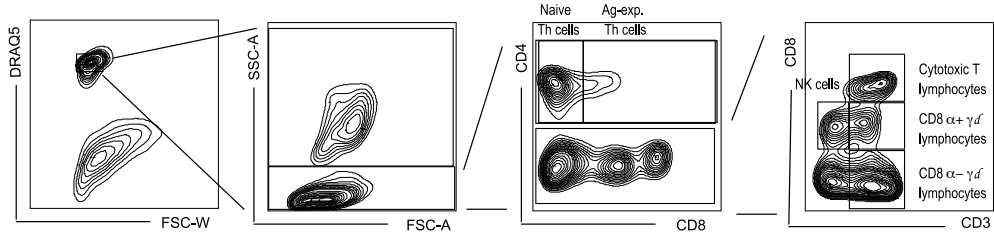

Supplement: Supplementary file 3 — Figure S2. Gating strategy for non B-lymphocyte subsets analysis. Representative flow cytometry profile is shown. Nucleated single cells were identified as DRAQ5-positive cells. Among SSC-Alo cells, CD4/CD8α/CD3 co-staining allowed the determination of 6 lymphocyte subsets as mentioned on the plots. (PDF 935 kb) [file 12917_2019_1809_MOESM3_ESM.pdf]
