# Supplementary material for: Effects of divergent selection upon adrenocortical activity on immune traits in pig
Source: BMC Vet Res. 2019 Mar 4;15:71. doi: 10.1186/s12917-019-1809-9 (PMC6398250; doi:10.1186/s12917-019-1809-9)
Supplement: Supplementary file 2 — Table S1. Variable’s contributions to the first two dimensions of the PCA. Variables that contributed to more than 10% to one axis are underlined. (PDF 35 kb) [file 12917_2019_1809_MOESM2_ESM.pdf]

Variables	Dim 1	Dim 2
Red blood cells	7,7	14,5
Hemoglobin	7,7	15,4
Hematocrit	5,5	18,4
Reticulocytes	6,4	2,3
Immature reticulocyte fraction	8,9	1,4
Platelets	0,5	8,4
Neutrophils	1,0	6,1
Monocytes	5,6	5,0
Naive Th lymphocytes	10,5	0,0
Ag-exp Th lymphocytes	6,7	1,0
CD8α- γδ T lymphocytes	1,3	1,9
Cytotoxic T lymphocytes	14,7	0,9
CD8α+ γδ T lymphocytes	4,9	12,3
NK cells	8,8	5,1
B lymphocytes	6,5	1,9
LPS-induced IL-8 secretion	0,0	1,8
LPS-induced TNFα secretion	0,1	0,0
LPS-induced IL-10 secretion	2,7	2,1
% phagocytes in mononuclear cells	0,2	0,5
% phagocytes in polymorphonuclear cells	0,0	1,0
