# Supplementary material for: Effects of divergent selection upon adrenocortical activity on immune traits in pig
Source: BMC Vet Res. 2019 Mar 4;15:71. doi: 10.1186/s12917-019-1809-9 (PMC6398250; doi:10.1186/s12917-019-1809-9)
Supplement: Supplementary file 4 — Table S2. List of genes whose expression was significantly modulated after LPS injection compared to their t0 level. (PDF 43 kb) [file 12917_2019_1809_MOESM4_ESM.pdf]

Gene	Name	Genbank Id	t1	t4	t24
ALOX15	arachidonate 15-lipoxygenase	396971	-		
ALOX5AP	arachidonate 5-lipoxygenase activating protein	397392	+	-	
C2H19orf59	MCEMP1 mast cell expressed membrane protein 1	100511550	+	-	-
CARD6	caspase recruitment domain family member 6	100627238	+		
CCL4	C-C motif chemokine ligand 4	396668	-	-	
CD24*	CD24 molecule	Not available	-	-	
CERS4	ceramide synthase 4	100127167		-	
CHI3L1	chitinase 3 like 1	396865			-
CNDP2	carnosine dipeptidase 2	100158127	+	-	
CSF2RA	colony stimulating factor 2 receptor alpha subunit	100620339		-	
DUSP2	dual specificity phosphatase 2	100622812	-		
FAS	Fas cell surface death receptor	396826		-	
GNG10	G protein subunit gamma 10	100515992		-	
JAK2	Janus kinase 2	397201		-	
CXCL8	C-X-C motif chemokine ligand 8	396880	-		
MEGF9	multiple EGF like domains 9	100157028	-		
MXD1	MAX dimerization protein 1	100625349	-	-	
PADI4	peptidyl arginine deiminase 4	100524749	-		
PDPN	podoplanin	100738269	+		
PSAP	prosaposin	100153167	+		
RAB31	RAB31, member RAS oncogene family	100737127	+	+	-
RGS2	regulator of G protein signaling 2	733670		-	
S100A12	S100 calcium binding protein A12	100301483	-	-	
S100A9	S100 calcium binding protein A9	100127489	-	-	
SCARB1	scavenger receptor class B member 1	397018	-		
SLA	Src like adaptor	100156099	-	-	
SOD2	superoxide dismutase 2	100154319		-	
STEFINA8	stefin A8	396867	-	-	
TANK	TRAF family member associated NFKB activator	100156366		-	
TIAM1	T cell lymphoma invasion and metastasis 1	100524587	+		
TMBIM6	transmembrane BAX inhibitor motif containing 6	396907		-	
TNFAIP6	TNF alpha induced protein 6	100286870	-	-	
VNN2	vanin 2	100153984	+		-
XAF1	XIAP associated factor 1	102159947	+		

* Sequence ID: XR_002343684.1
